# Supplementary material for: AMPA receptor anchoring at CA1 synapses is determined by N-terminal domain and TARP γ8 interactions
Source: Nat Commun. 2021 Aug 23;12:5083. doi: 10.1038/s41467-021-25281-4 (PMC8382838; doi:10.1038/s41467-021-25281-4)
Supplement: Supplementary file 1 — Supplementary Information [file 41467_2021_25281_MOESM1_ESM.pdf]

Supplementary Information for

AMPA receptor anchoring at CA1 synapses is determined by N-terminal domain and TARP γ8 interactions

Jake F. Watson†, Alexandra Pinggera†, Hinze Ho and Ingo H. Greger*

† these authors contributed equally; * for correspondence: ig@mrc-lmb.cam.ac.uk

Containing:

Supplementary Tables 1-3

Supplementary Figures 1-7

Supplementary Table 1: Dual Cell Synaptic Current Properties - GluA2Q data

	Rectification Index			
Construct	Untrans.	Transf.	n (pairs)	p*
GluA2Q	0.61 ± 0.05	0.22 ± 0.03	6	0.0009
GluA2Q ΔNSF	0.62 ± 0.04	0.19 ± 0.028	11	<0.0001
GluA2Q ΔPDZ	0.60 ± 0.08	0.24 ± 0.04	8	0.0008
GluA2Q A1ICD	0.49 ± 0.05	0.17 ± 0.03	12	<0.0001
GluA2Q CTD-null	0.62 ± 0.04	0.19 ± 0.03	15	<0.0001
GluA2Q ΔNTD	0.60 ± 0.04	0.21 ± 0.03	9	<0.0001
GluA2Q ΔNTD ΔNSF	0.56 ± 0.05	0.38 ± 0.03	10	0.013
GluA2Q ΔNTD ΔPDZ	0.57 ± 0.04	0.27 ± 0.03	12	<0.0001
GluA2Q ΔNTD A1ICD	0.56 ± 0.05	0.41 ± 0.03	9	0.018
GluA2Q ΔNTD CTD-null	0.61 ± 0.02	0.15 ± 0.02	15	<0.0001

	AMPAR EPSC Amplitudes (pA)			
Construct	Untrans.	Transf.	n (pairs)	p*
GluA2Q	37.4 ± 10.2	54.8 ± 10.2	8	0.0078
GluA2Q ΔNSF	34.8 ± 3.9	48.2 ± 7.5	11	0.024
GluA2Q ΔPDZ	23.1 ± 2.9	34.7 ± 5.3	9	0.0039
GluA2Q A1ICD	39.1 ± 8.0	54.6 ± 9.1	12	0.043
GluA2Q CTD-null	53.1 ± 6.3	65.7 ± 8.5	20	0.030
GluA2Q ΔNTD	40.6 ± 4.1	21.8 ± 2.2	14	0.0001
GluA2Q ΔNTD ΔNSF	37.6 ± 3.2	29.4 ± 4.5	11	0.024
GluA2Q ΔNTD ΔPDZ	91.4 ± 15.5	65.8 ± 11.0	12	0.016
GluA2Q ΔNTD A1ICD	38.6 ± 5.0	31.7 ± 6.1	9	0.049
GluA2Q ΔNTD CTD-null	50.0 ± 4.5	30.2 ± 3.6	15	0.0002

* Statistical analysis - Rectification Index: two-tailed paired t-test; EPSC ampltiudes: two-tailed Wilcoxon matched-pairs signed rank test.

Supplementary Table 2: Dual Cell Synaptic Current Properties - GluA1 data

	Rectification Index			
Construct	Untrans.	Transf.	n (pairs)	p*
GluA1	0.53 ± 0.03	0.37 ± 0.03	7	0.0058
GluA1 ΔPDZ	0.60 ± 0.04	0.38 ± 0.04	13	<0.0001
GluA1 ΔPDZ +tCKII	0.58 ± 0.06	0.41 ± 0.05	12	0.012
GluA1 CTD-null	0.58 ± 0.04	0.45 ± 0.04	17	0.0016
GluA1 + TTX incubation	0.64 ± 0.07	0.31 ± 0.03	7	0.0082
GFP-GluA1	0.60 ± 0.04	0.54 ± 0.04	16	0.086
GluA1 ΔNTD	0.57 ± 0.07	0.53 ± 0.05	9	0.54
GluA1 ΔNTD ΔPDZ	0.52 ± 0.05	0.49 ± 0.04	9	0.41
GluA1 ΔNTD +tCKII	0.56 ± 0.05	0.45 ± 0.05	10	0.0083
GluA1 ΔNTD ΔPDZ + tCKII	0.63 ± 0.03	0.60 ± 0.05	8	0.70

	AMPAR EPSC Amplitudes (pA)			
Construct	Untrans.	Transf.	n (pairs)	p*
GluA1	24.5 ± 1.4	19.6 ± 1.8	7	0.078
GluA1 ΔPDZ	60.8 ± 7.2	56.4 ± 8.2 pA	14	0.30
GluA1 ΔPDZ +tCKII	50.8 ± 9.0	106.2 ± 16.0	8	0.0078
GluA1 CTD-null	46.8 ± 5.8	37.9 ± 3.8	17	0.38
GluA1 + TTX incubation	72.3 ± 12.7	52.2 ± 5.6	8	0.15
GFP-GluA1	65.3 ± 5.7	49.8 ± 5.4	13	0.13
GluA1 ΔNTD	29.5 ± 4.9	19.1 ± 2.3	9	0.055
GluA1 ΔNTD ΔPDZ	59.2 ± 6.1	47.5 ± 4.4	9	0.30
GluA1 ΔNTD +tCKII	49.9 ± 6.8	43.1 ± 12.0	9	0.13
GluA1 ΔNTD ΔPDZ + tCKII	21.7 ± 2.7	38.8 ± 4.7	16	0.0016

* Statistical analysis - Rectification Index: two-tailed paired t-test; EPSC ampltiudes: two-tailed Wilcoxon matched-pairs signed rank test.

Supplementary Table 3: List of Primers used for plasmid generation

Name	Sequence	Notes
scFv-Clasp light chain forward primer	5’-TAGTAGTAACCGGTCATATGGAAGTGAAG-TTGGTTGAATCTG-3’	inserting AgeI site
scFv-Clasp light chain reverse primer	5’-TAGTAGTAGGTACCCTTAGCCTCTATGGC-ATCCAGG-3’	Inserting KpnI site
scFv-Clasp heavy chain forward primer	5’-TAGTAGTAACCGGTATGGACATCGAATTG-ACTCAGTCTCC-3’	inserting AgeI site
scFv-Clasp heavy chain reverse primer	5’-TAGTAGTATGTACATTACTTAGCCTCTAT-GGCATCCA-GG-3’	Inserting Bsp1407I site

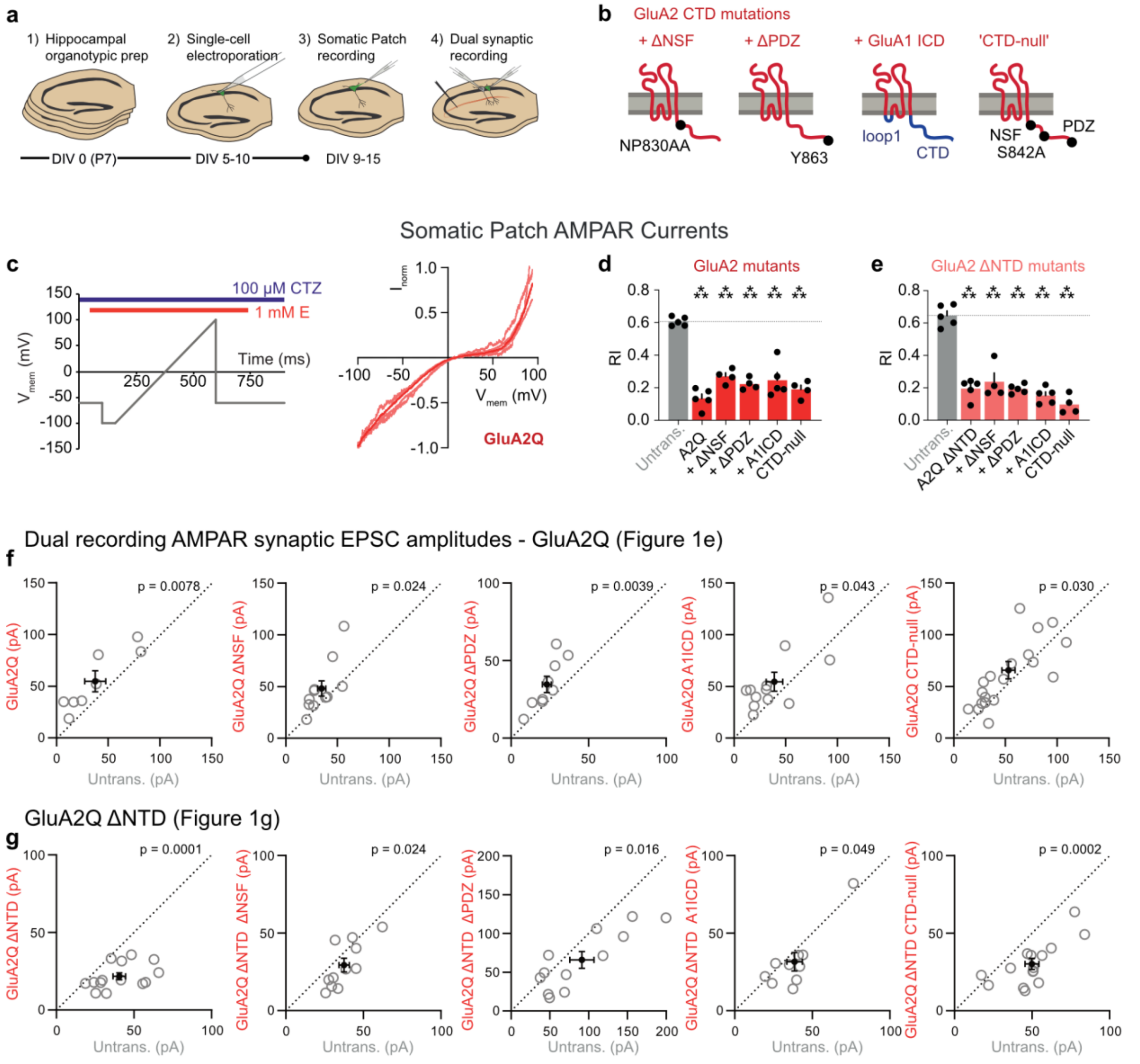

Supplementary Figure 1. Somatic and Synaptic recordings on GluA2Q expression. a Approach for analysis of mutant AMPAR constructs. **b** Construct schematics depicting the locations of CTD mutations on GluA2 ΔNTD. Peptide topology is displayed relative to the membrane (grey). **c** Somatic AMPAR currents demonstrate surface trafficking for GluA2 mutations by rectification index measurements. Protocol for surface rectification measurement by glutamate (E) and cyclothiazide (CTZ) application during a membrane voltage ramp (left). Representative glutamate gated currents for GluA2 overexpressing neurons (right, individual cells - light, average response - bold), normalised to -100 mV amplitude. **d** Somatic patch rectification index on overexpression of CTD mutation constructs of GluA2Q (Untrans.: 0.61 ± 0.01, n = 5; GluA2Q: 0.13 ± 0.03, n = 5; GluA2Q ΔNSF: 0.27 ± 0.02, n = 4; GluA2Q ΔPDZ: 0.22 ± 0.02, n = 4; GluA2Q A1ICD: 0.25 ± 0.05, n = 5; GluA2Q CTD-null: 0.19 ± 0.03, n = 4; [$F(5, 21) = 36.38$, $p<0.0001$], One-way ANOVA with Dunnett's multiple comparisons test, bars

represent mean values), and **e,** GluA2Q ΔNTD (Untrans.: 0.65 ± 0.03, n = 5; GluA2Q ΔNTD: 0.20 ± 0.03, n = 5; GluA2Q ΔNTD ΔNSF: 0.24 ± 0.05, n = 4; GluA2Q ΔNTD ΔPDZ: 0.19 ± 0.01, n = 5; GluA2Q ΔNTD A1ICD: 0.15 ± 0.02, n = 5; GluA2Q ΔNTD CTD-null: 0.10 ± 0.03, n = 4; [$F(5, 22) = 46.22$, $p<0.0001$], One-way ANOVA with Dunnett's multiple comparisons test, bars represent mean values). **f-g** Scatters of dual synaptic EPSC amplitudes for normalized data presented in **Figure 1e** and **1g**. Filled circles represent mean ± SEM. Data values are presented in **Supplementary Table 1**. Source data are provided as a Source Data file.

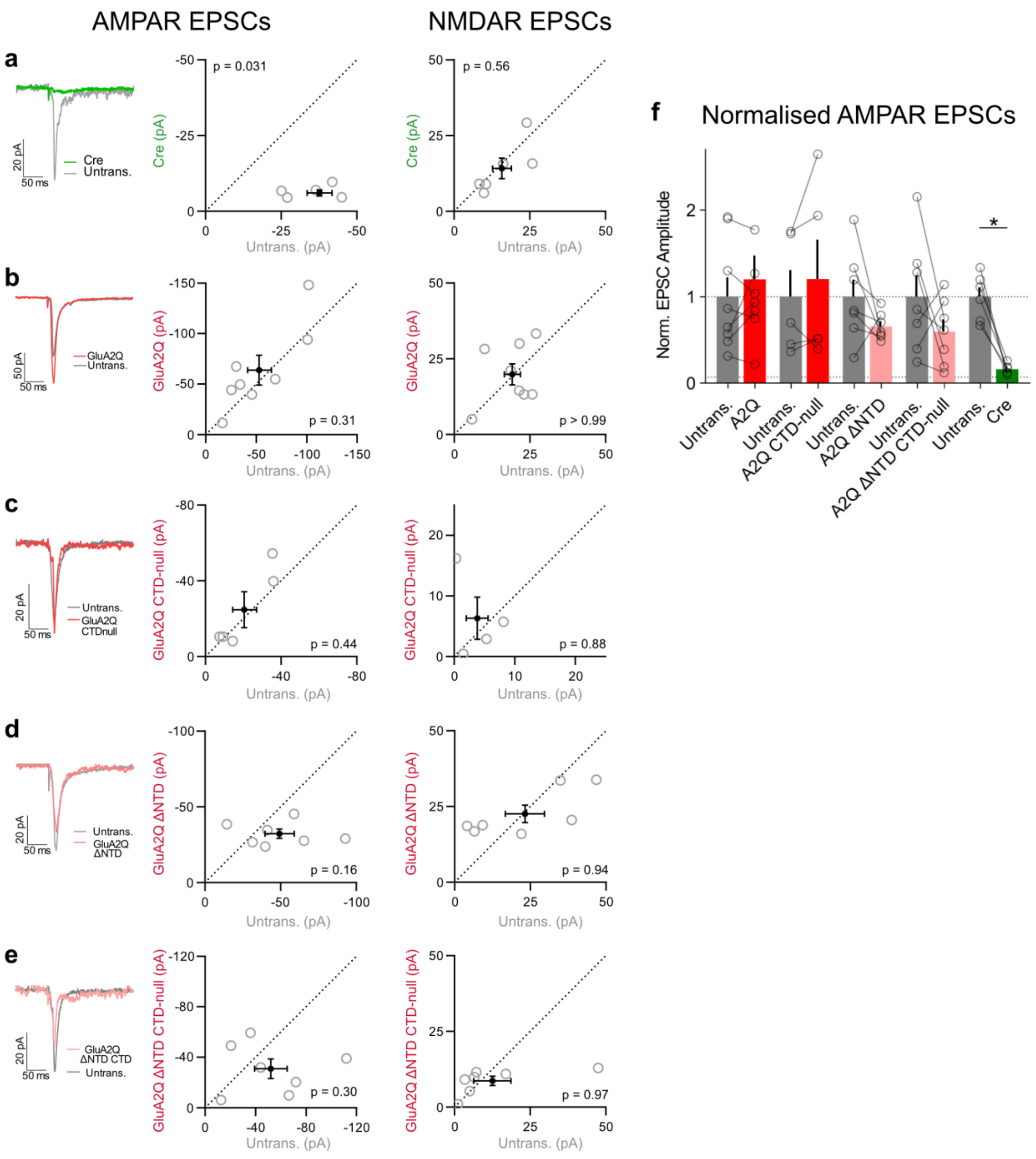

Supplementary Figure 2. Recovery of AMPAR-null transmission by CTD-null receptors. a Expression of Cre-recombinase in Gria1-3fl tissue abolished AMPAR, but not NMDAR synaptic transmission (AMPAR EPSCS (pA) - Untransduced (Untrans): 37.6 ± 4.1 pA, Cre: 6.1 ± 0.9 pA, n=6 pairs, p=0.031. NMDAR EPSCS (pA) – Untrans.: 15.8 ± 3.1 pA, Cre: 1.2 ± 3.4 pA, n=6, p=0.56). **b-e** GluA2Q and GluA2Q ΔNTD with and without CTD interaction site mutagenesis (CTD-null) can rescue AMPAR EPSCs, demonstrating no requirement for the CTD in synaptic anchoring (GluA2Q - Untrans.: 53.1 ± 11.9 pA, Rescue: 63.8 ± 14.7 pA, n = 8 pairs, p=0.031. GluA2Q CTD-null – Untrans.: 20.6 ± 6.3 pA, Rescue: 24.7 ± 9.4 pA, n = 5 pairs, p=0.44. GluA2Q ΔNTD – Untrans.: 49.2 ± 9.7 pA, Rescue: 32.3

± 2.9 pA, n = 7 pairs, p=0.16. GluA2Q ΔNTD CTD-null – Untrans.: 52.1 ± 13.0 pA, Rescue: 30.9 ± 7.5 pA, n = 7 pairs, p=0.30), while NMDAR EPSCs were unaffected (GluA2Q - Untrans.: 19.2 ± 2.6 pA, Rescue: 19.9 ± 3.5 pA, n = 8 pairs, p=0.56. GluA2Q CTD-null – Untrans.: 3.8 ± 1.8 pA, Rescue: 6.3 ± 3.5 pA, n = 4 pairs, p>0.99. GluA2Q ΔNTD – Untrans.: 23.2 ± 6.5 pA, Rescue: 22.6 ± 2.9 pA, n = 7 pairs, p=0.88. GluA2Q ΔNTD CTD-null – Untrans.: 12.6 ± 6.1 pA, Rescue: 8.7 ± 1.6 pA, n = 7 pairs, p=0.97). Filled circles represent mean ± SEM. **f** Data normalized to Untransfected EPSC amplitudes demonstrates that CTD modification does not strongly influence rescue by either GluA2Q or GluA2Q ΔNTD. Bars represent mean values + SEM. For n-numbers and p-values see panels a-e, * indicates p<0.05. All statistical analysis (**S2 a-f**) were performed with a Wilcoxon matched-pairs signed rank test. Source data are provided as a Source Data file.

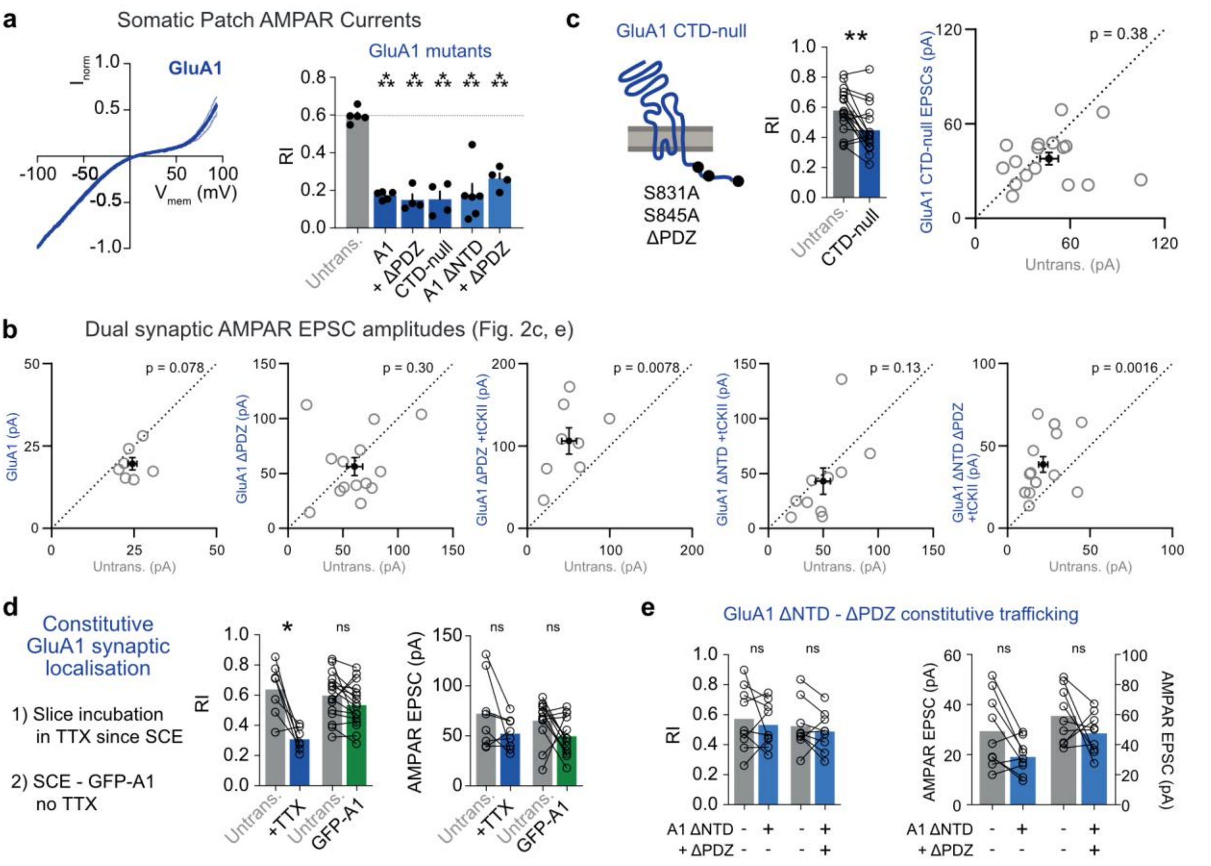

Supplementary Figure 3. Somatic and Synaptic recordings on expression of GluA1. a Somatic AMPAR currents demonstrate surface trafficking for GluA1 mutations by rectification index measurements GluA1 (Untrans.: 0.60 ± 0.02, n = 5; GluA1: 0.17 ± 0.01, n = 5; GluA1 ΔPDZ: 0.15 ± 0.03, n = 4; GluA1 CTD-null: 0.15 ± 0.04; GluA1 ΔNTD: 0.18 ± 0.06, n = 6; GluA1 ΔNTD ΔPDZ: 0.26 ± 0.03, n = 4; [F(5, 22) = 21.73, p<0.0001], One-way ANOVA with Dunnett's multiple comparisons test). **b** Synaptic EPSC amplitudes for normalized data in **Figures 2c** and **2e**. **c** Schematic and synaptic recordings demonstrating synaptic localisation of GluA1 CTD-null receptors (RI: two—tailed paired t-test, p=0.0016, AMPAR EPSCs: two-tailed Wilcoxon matched-pairs signed rank test, p=0.38). **d** GluA1 constitutive synaptic localization does not require ongoing slice activity, as RI is altered even which activity is blocked by 1 µM TTX application from the day of transfection. Synaptic localization of GluA1 is prevented by EGFP-tagging at the N-terminus. **e** GluA1 ΔNTD does not contribute to synaptic transmission regardless of PDZ_{GluA1} interactions (Wilcoxon matched-pairs signed rank test). Data values are presented in **Supplementary Table 2**. * indicates p<0.05, ** p<0.01, *** p<0.001 and ^{ns} specifies no significance. Bars in panels a, c-e represent mean values, filled circles in b-c represent mean ± SEM. Source data are provided as a Source Data file.

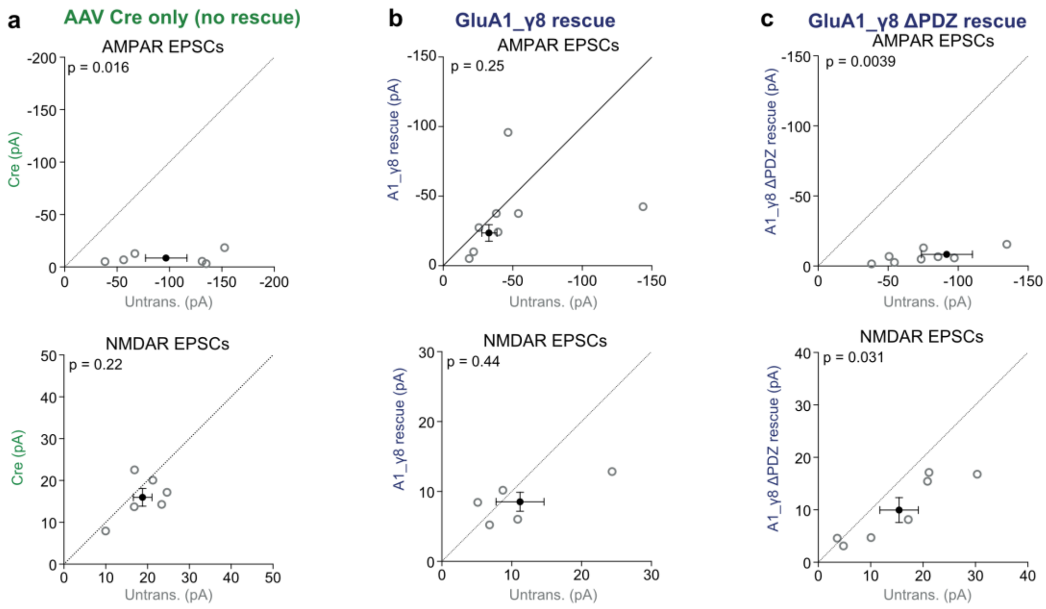

Supplementary Figure 4. Supporting data for knockout and rescue of GluA1_ γ8 receptors. a-c Scatter graphs of synaptic AMPAR and NMDAR EPSC amplitudes between Cre-transduced and untransduced (Untrans.) neurons (**a** - AMPAR EPSCs - Untrans.: -86.5 ± 19.6 pA, Cre: -7.9 ± 2.2 pA, n = 7 pairs, p=0.016; NMDAR EPSCs - Untrans.: 22.7 ± 5.4 pA, Cre: 18.3 ± 3.3 pA, n = 6 pairs, p=0.22), control and GluA1_γ8 rescued pairs (**b -** AMPAR EPSCs - Untrans.: -48.8 ± 14.3 pA, GluA1_γ8 rescue: -35.1 ± 9.9 pA, n = 8 pairs, p=0.25; NMDAR EPSCs - Untrans.: 11.2 ± 3.4 pA, GluA1_γ8 rescue: 8.6 ± 1.4 pA, n = 5 pairs, p=0.44), and control and GluA1_γ8 ΔPDZ rescued pairs (**c -** AMPAR EPSCs - Untrans.: -91.8 ± 18.2, GluA1_γ8 ΔPDZ: -8.5 ± 2.0, n = 9 pairs, p=0.0039; NMDAR EPSCs - Untrans.: 15.5 ± 3.7 pA, GluA1_γ8 ΔPDZ: 10.0 ± 2.4 pA, n = 7 pairs, p=0.031). All statistics performed with a Wilcoxon matched-pairs signed rank test, filled circles represent mean ± SEM. Source data are provided as a Source Data file.

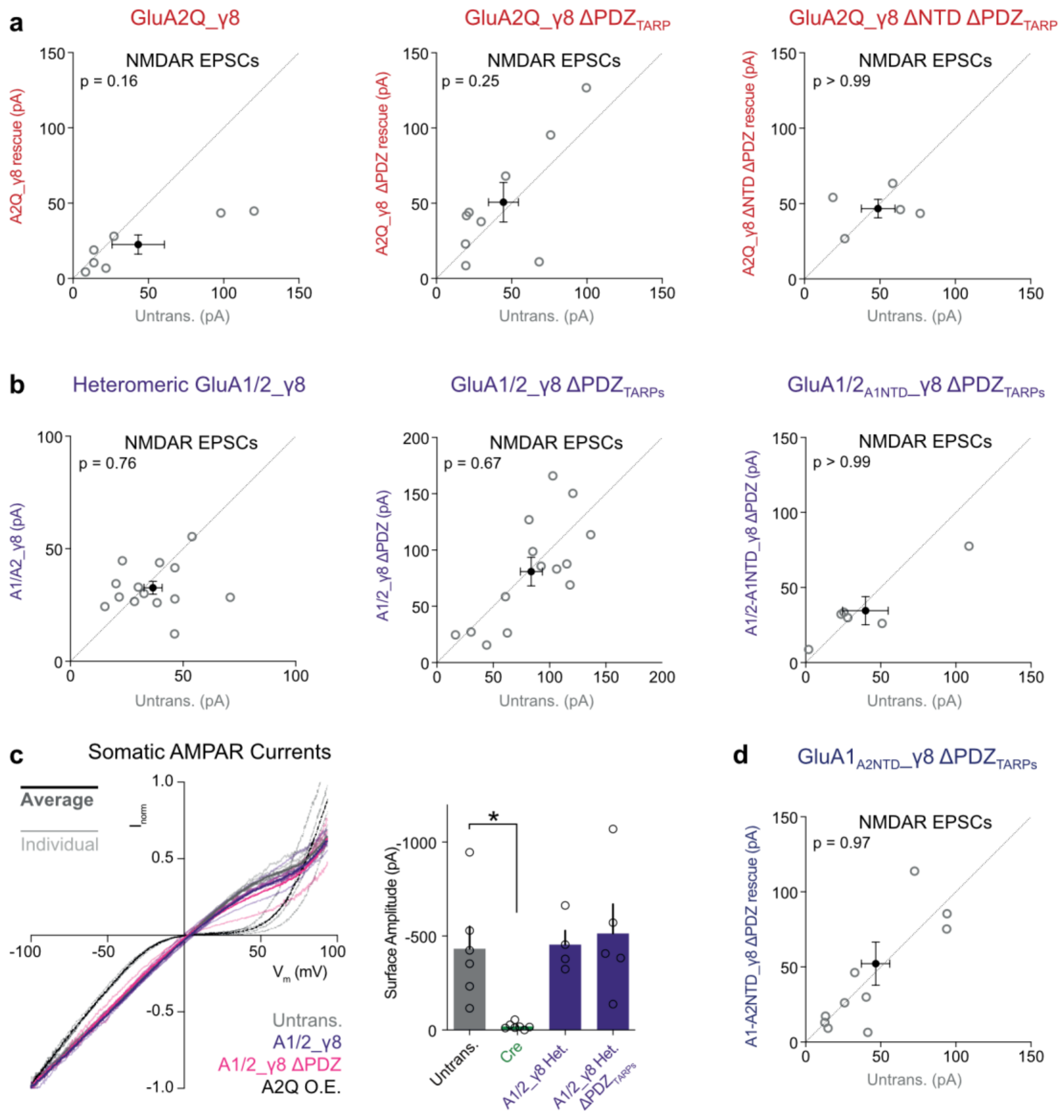

Supplementary Figure 5. Supporting figures for knockout and rescue using GluA2_γ8 or heteromeric AMPARs. a Synaptic NMDAR currents are unchanged after AMPAR knockout and rescue with different GluA2Q_γ8 constructs (GluA2Q_γ8 - Untrans.: 43.4 ± 17.3 pA, Rescue: 22.5 ± 6.4 pA, n = 7 pairs, p=0.16; GluA2Q_γ8 ΔPDZ - Untrans.: 44.6 ± 10.0 pA, Rescue: 50.8 ± 13.2 pA, n = 9 pairs, p=0.25; GluA2Q_γ8 ΔPDZ ΔNTD - Untrans.: 48.9 ± 11.1 pA, Rescue: 46.8 ± 6.1 pA, n = 5 pairs, p>0.99, All tests are Wilcoxon matched-pairs signed rank tests). **b** Synaptic NMDAR currents are unchanged after AMPAR knockout and rescue with different tandem heteromeric receptors (A1_γ8/A2_γ8 - Untrans.: 36.7 ± 4.1 pA, Rescue: 32.7 ± 2.9 pA, n = 14 pairs, p=0.76; A1_γ8/A2_γ8 ΔPDZs - Untrans.: 83.9 ± 9.7 pA, Rescue: 81.0 ± 12.8 pA, n = 14 pairs, p=0.67; A1_γ8/$A2_{A1NTD}$_γ8 ΔPDZs - Untrans.: 39.8 ± 15.2 pA, rescue: 34.7 ± 9.3 pA, n = 6 pairs, p>0.99, All tests are Wilcoxon matched-pairs signed rank tests). **c** Somatic AMPAR currents from outside-out patches on heteromeric receptor rescue. left -

Normalised current traces from holding potential ramp application to patches in the presence of glutamate, demonstrating non-rectifying responses on heteromeric receptor rescue. Both cell-averaged responses (bold) and individual cells (transparent) are shown (untransfected cells - grey, A1_γ8/A2_γ8 rescue - purple, A1_γ8/A2_γ8 ΔPDZs rescue - pink) against strongly rectifying GluA2Q overexpressing cell responses (black) for reference. right **-** Current amplitudes from somatic patches demonstrate abolishment of AMPAR response on Cre transduction, and similar surface receptor rescue by heteromeric receptors with and without TARP PDZ interactions (Untrans.: -434 ± 118 pA, n = 6; Cre: -20 ± 6.5 pA, n = 7; A1_γ8/A2_γ8 rescue: -456 ± 74 pA, n = 4; A1_γ8/A2_γ8 ΔPDZs rescue: -515 ± 155 pA, n = 5; one-way ANOVA with Dunnett' multiple comparisons test [$F(3,18) = 6.007$, $p=0.0051$]). * indicates $p<0.05$. **d** NMDAR currents are unchanged on rescue with $GluA1_{A2NTD}$_γ8 ΔPDZs (Untrans.: 46.7 ± 9.4 pA, Rescue: 52.3 ± 14.5, n = 11 pairs, $p=0.96$, Wilcoxon matched-pairs signed rank test). All data shown as mean ± SEM. Source data are provided as a Source Data file.

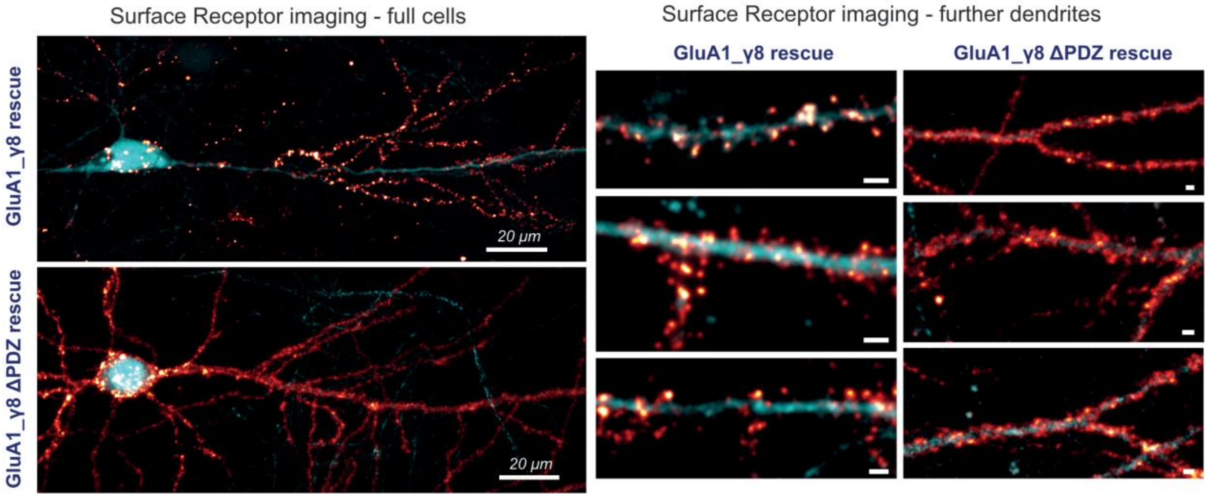

Supplementary Figure 6. Additional images supporting Figure 4. Further representative images of surface AMPARs cell distribution from individual cells on GluA1_γ8 rescue ± PDZ_{TARP} interactions (cyan - tdTomato cell filler, red/glow - Streptavidin647 labelled surface AMPARs). For details of cell numbers and repetitions see Figure 4. Dendrite scale bars = 4 μm.

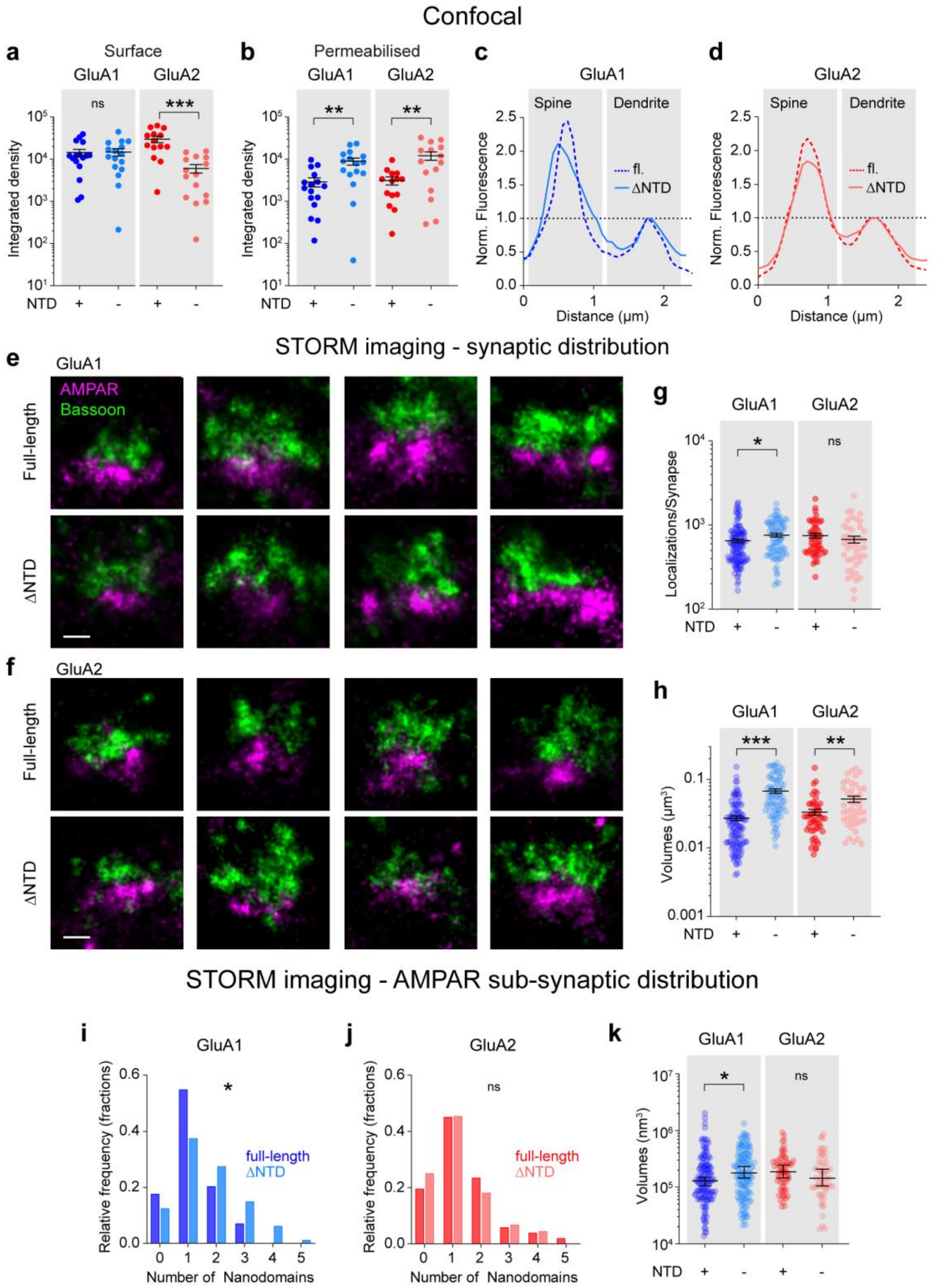

Supplementary Figure 7. Supporting data for confocal and STORM imaging of cultured hippocampal neurons. a Surface expression (confocal imaging) was unaltered for GluA1 ΔNTD receptors (integrated densities, left, GluA1 full-length: 14410 ± 2817, 16 cells, GluA1 ΔNTD: 14880 ± 2891; 16 cells, 5 independent preparations; p=0.9080, two-tailed unpaired t-test) but significantly reduced for GluA2 ΔNTD receptors (integrated densities, right GluA2 full-length: 29855 ± 5081, 14 cells,

GluA2 ΔNTD: 6072 ± 1360; 15 cells, 4 independent preparations; $p<0.0001$, two-tailed unpaired t-test) compared to respective full-length controls. **b** NTD deletion resulted in increased intracellular expression levels for both GluA1 (integrated densities, left, GluA1 full-length: 4007 ± 956.1, GluA1 ΔNTD: 12403 ± 2352; n-numbers given in a; $p=0.0025$, two-tailed unpaired t-test) and GluA2 (right, GluA2 full-length: 4295 ± 923.4, GluA2 ΔNTD: 16793 ± 3806; n-numbers given in a; $p=0.0046$, two-tailed unpaired t-test) compared to full-length receptors. **c-d** Representative line-scans across spines and dendrites for full-length and NTD-deleted GluA1 (**c**) and GluA2 (**d**) receptors (See **Figure 5**). **e-f** Representative 3D STORM images to demonstrate synaptic distributions of presynaptic marker bassoon (green) and post-synaptic GluA1 (e) and GluA2 (f) full-length and ΔNTD receptors (magenta) (Scale bars: 100 nm). **g** NTD deleted GluA1 (left, GluA1 full-length: 647.5 ± 33.8, GluA1 ΔNTD 760.6 ± 39.5; $p=0.0190$, two-tailed unpaired t-test) but not ΔNTD GluA2 receptors (right, GluA2 full-length: 749.9 ± 50.4, GluA2 ΔNTD: 675.4 ± 64.9; $p=0.0838$, two-tailed unpaired t-test) displayed more localisations per synapse upon 3D STORM imaging. **h** NTD deleted GluA1 and GluA2 receptors adopted larger synaptic volumes compared to respective full-length receptors (synaptic volumes (μm^3) left, GluA1 full-length: 0.027 ± 0.002, GluA1 ΔNTD 0.067± 0.005; $p<0.0001$, two-tailed unpaired t-test; (right, GluA2 full-length: 0.033 ± 0.003, GluA2 ΔNTD: 0.052 ± 0.005; $p=0.0038$, two-tailed unpaired t-test). **i-j** Relative frequency distribution of nanocluster numbers across individual synapses was changed in GluA1 ΔNTD receptors (left, $p=0.017$, Kolmogorov-Smirnov test) but unaffected in NTD-lacking GluA2 receptors (right, $p>0.999$, Kolmogorov-Smirnov test). **k** NTD deleted GluA1 (nanodomain volumes (nm^3) left, GluA1 full-length: $1.30*10^5$ ± $1.06*10^5$-$1.50*10^5$, GluA1 ΔNTD $1.79*10^5$ ± $1.45*10^5$-$2.33*10^5$; $p=0.0307$, two-tailed Mann-Whitney test) but not ΔNTD GluA2 receptors (right, GluA2 full-length: $1.88*10^5$ ± $1.45*10^5$-$2.46*10^5$, GluA2 ΔNTD: $1.44*10^5$ ± $1.05*10^5$-$2.09*10^5$; $p=0.2257$, two-tailed Mann-Whitney test) displayed larger nanodomain volumes. Data in panels a, b, g and h shown as mean ± SEM and in panel k as median ± lower-upper 95% CI, * indicates $p<0.05$, ** $p<0.01$, *** $p<0.001$ and ^{ns} specifies no significance. n-numbers for STORM data are given in legend to **Figures 5&6**. Source data are provided as a Source Data file.
