# Supplementary material for: AMPA receptor anchoring at CA1 synapses is determined by N-terminal domain and TARP γ8 interactions
Source: Nat Commun. 2021 Aug 23;12:5083. doi: 10.1038/s41467-021-25281-4 (PMC8382838; doi:10.1038/s41467-021-25281-4)
Supplement: Supplementary file 2 — Reporting Summary [file 41467_2021_25281_MOESM2_ESM.pdf]

nature portfolio

Corresponding author(s): Ingo H. Greger

Last updated by author(s): Jul 12, 2021

Reporting Summary

Nature Portfolio wishes to improve the reproducibility of the work that we publish. This form provides structure for consistency and transparency in reporting. For further information on Nature Portfolio policies, see our Editorial Policies and the Editorial Policy Checklist.

Statistics

For all statistical analyses, confirm that the following items are present in the figure legend, table legend, main text, or Methods section.

n/a	Confirmed	
☐	☒	The exact sample size (n) for each experimental group/condition, given as a discrete number and unit of measurement
☐	☒	A statement on whether measurements were taken from distinct samples or whether the same sample was measured repeatedly
☐	☒	The statistical test(s) used AND whether they are one- or two-sided<br>*Only common tests should be described solely by name; describe more complex techniques in the Methods section.*
☒	☐	A description of all covariates tested
☐	☒	A description of any assumptions or corrections, such as tests of normality and adjustment for multiple comparisons
☐	☒	A full description of the statistical parameters including central tendency (e.g. means) or other basic estimates (e.g. regression coefficient) AND variation (e.g. standard deviation) or associated estimates of uncertainty (e.g. confidence intervals)
☐	☒	For null hypothesis testing, the test statistic (e.g. F, t, r) with confidence intervals, effect sizes, degrees of freedom and P value noted<br>*Give P values as exact values whenever suitable.*
☒	☐	For Bayesian analysis, information on the choice of priors and Markov chain Monte Carlo settings
☒	☐	For hierarchical and complex designs, identification of the appropriate level for tests and full reporting of outcomes
☒	☐	Estimates of effect sizes (e.g. Cohen's d, Pearson's r), indicating how they were calculated

Our web collection on statistics for biologists contains articles on many of the points above.

Software and code

Policy information about availability of computer code

Data collection	All data were collected using commercial software: electrophysiology data was acquired using Clampex (pClamp10), confocal images were acquired with Leica Application Suite X (LAS X) 3.7.2 software and STORM images were collected using with NIS Elements Advanced Research software 4.13 (Nikon)
Data analysis	Electrophysiology data was analysed with Clampfit (pClamp 10 - Molecular Devices), confocal images were analysed using Fiji (ImageJ 1.52v), STORM images were reconstructed with NIS Elements Advanced Research software 4.13 (Nikon) and analysed with customised published/ freely available code in MATLAB R2019b. Code was customised for purpose from published analysis scripts. Scripts are available here: http:// chemometria.us.edu.pl/index.php?goto=downloads, https://github.com/alexgkendall/OPTICS_Clustering.<br>All statistical analysis was performed in GraphPad Prism 8.

For manuscripts utilizing custom algorithms or software that are central to the research but not yet described in published literature, software must be made available to editors and reviewers. We strongly encourage code deposition in a community repository (e.g. GitHub). See the Nature Portfolio guidelines for submitting code & software for further information.

Data

Policy information about availability of data

All manuscripts must include a data availability statement. This statement should provide the following information, where applicable:

- Accession codes, unique identifiers, or web links for publicly available datasets
- A description of any restrictions on data availability
- For clinical datasets or third party data, please ensure that the statement adheres to our policy

Data supporting the findings of this study are summarized in the Source Data File.

Field-specific reporting

Please select the one below that is the best fit for your research. If you are not sure, read the appropriate sections before making your selection.

- [x] Life sciences
- [ ] Behavioural & social sciences
- [ ] Ecological, evolutionary & environmental sciences

For a reference copy of the document with all sections, see nature.com/documents/nr-reporting-summary-flat.pdf

Life sciences study design

All studies must disclose on these points even when the disclosure is negative.

Sample size	No sample size calculation was performed as effect sizes were unknown prior to the study. Sample sizes were estimated based on previous experience with data of this type (see Watson et al. Elife 2017) and are in line with similar studies (see Diaz-Alonso et al. PNAS 2017). Presented sample sizes were deemed sufficient based on the reproducibility of the results across several independent preparations.
Data exclusions	For STORM imaging only neurons with similar expression levels of deltaNTD versus respective full-length receptors were included in the study to avoid bias in nanocluster detection/quantification based on differences in surface expression. Moreover, only synapses with an unambiguous orientation (i.e. pre- versus postsynaptic), a density (localisations/area) greater than 3x10^3 and a synaptic area < 0.6 µm2 were incorporated into the analysis to ensure accurate determination of the synaptic and sub-synaptic organisation of the investigated proteins, high labelling density and the inclusion of individual synapses only, respectively. All these exclusion criteria were pre-established. Electrophysiology data were excluded following predetermined standard quality control criteria (recording stability, holding current).
Replication	The majority of the datasets were replicated 2-3 times and all replications were successful.
Randomization	Randomisation is not relevant to this study as samples were not divided into experimental groups.
Blinding	For the majority of experiments, investigators were not blinded during data acquisition due to the technical complication of doing so when performing challenging and multi-step experimental procedures, for which only one experimenter was sufficiently trained in each case. A small subset of electrophysiology experiments were repeated blinded to confirm findings.

Reporting for specific materials, systems and methods

We require information from authors about some types of materials, experimental systems and methods used in many studies. Here, indicate whether each material, system or method listed is relevant to your study. If you are not sure if a list item applies to your research, read the appropriate section before selecting a response.

Materials & experimental systems

n/a	Involved in the study	
☐	☒	Antibodies
☐	☒	Eukaryotic cell lines
☒	☐	Palaeontology and archaeology
☐	☒	Animals and other organisms
☒	☐	Human research participants
☒	☐	Clinical data
☒	☐	Dual use research of concern

Methods

n/a	Involved in the study	
☒	☐	ChIP-seq
☒	☐	Flow cytometry
☒	☐	MRI-based neuroimaging

Antibodies

Antibodies used	rabbit anti-HA (Sigma-Aldrich, Cat.# H6908, RRID:AB_260070), rabbit anti-bassoon (Synaptic Systems Cat# 141 003, RRID:AB_887697), goat anti-rabbit IgG AF568 (Thermo Fisher Scientific Cat# A-11036, RRID:AB_10563566), goat anti-rabbit IgG AF647 (Thermo Fisher Scientific Cat# A-21245, RRID:AB_2535813), single chain Fv fragment (scFv) against HA, based on clone 12CA5, kindly provided by Junichi Takagi, Institute for Protein Research, Laboratory of Protein Synthesis and Expression, Osaka University, Japan, for reference see Arimori T et al., Structure 217, DOI: 10.1016/j.str.2017.08.011.
Validation	anti-HA specificity and suitability for immunocytochemistry validated by supplier. In addition, our experiements demonstrate validity as no signal was observed in neurons lacking HA-tagged proteins for both rabbit anti-HA and scFv anti-HA. Rabbit anti-bassoon is specific to rat and mouse bassoon, specificity validated by supplier based on western blot reactivity, immunocytochemistry detection of protein distribution, and lack of reactivity in other species tissue (chicken). Suitable dilutions for all antibodies and scFv anti-HA were tested beforehand.

Eukaryotic cell lines

Policy information about cell lines

Cell line source(s)	HEK293S GnTI– cells (ATCC, Cat.# CRL-3022, RRID:CVCL_A785) were obtained from Radu Aricescu
Authentication	No further authentication was performed for cell lines in this study.
Mycoplasma contamination	No mycoplasma testing was performed for this study.
Commonly misidentified lines (See ICLAC register)	HEK cells are listed in the register, however they came from a reliable source and were only used for Fv-Clasp anti HA production and not for any functional studies.

Animals and other organisms

Policy information about studies involving animals; ARRIVE guidelines recommended for reporting animal research

Laboratory animals	This study used C57BL/6JOla wild-type (RRID:MGI:3691859) and Gria1-3fl/fl mice of both sexes at postnatal days 0-1 and 6-8. Animals were housed with unlimited access to food and water under a standard 12 hours light dark cycle at normal room temperature (20-22 degrees centigrade) at 45-65% humidity.
Wild animals	The study did not involve wild animals.
Field-collected samples	The study did not involve samples collected from the field.
Ethics oversight	All experimental procedures were performed under PPL P81248620 in accordance with UK Home Office regulations and were licensed under the Animals (Scientific Procedures) Act of 1986 following local (AWERB) ethical approval (Institute: MRC Laboratory of Molecular Biology).

Note that full information on the approval of the study protocol must also be provided in the manuscript.
